# Genetic variants associated with fasting blood lipids in the U.S. population: Third National Health and Nutrition Examination Survey

**DOI:** 10.1186/1471-2350-11-62

**Published:** 2010-04-20

**Authors:** Man-huei Chang, Ajay Yesupriya, Renée M Ned, Patricia W Mueller, Nicole F Dowling

**Affiliations:** 1 National Office of Public Health Genomics, Centers for Disease Control and Prevention, Atlanta, GA, USA; 2 National Center for Environmental Health, Centers for Disease Control and Prevention, Atlanta, GA, USA

## Abstract

**Background:**

The identification of genetic variants related to blood lipid levels within a large, population-based and nationally representative study might lead to a better understanding of the genetic contribution to serum lipid levels in the major race/ethnic groups in the U.S. population.

**Methods:**

Using data from the second phase (1991-1994) of the Third National Health and Nutrition Examination Survey (NHANES III), we examined associations between 22 polymorphisms in 13 candidate genes and four serum lipids: high-density lipoprotein cholesterol (HDL-C), low-density lipoprotein cholesterol (LDL-C), total cholesterol (TC), and triglycerides (TG). Univariate and multivariable linear regression and within-gene haplotype trend regression were used to test for genetic associations assuming an additive mode of inheritance for each of the three major race/ethnic groups in the United States (non-Hispanic white, non-Hispanic black, and Mexican American).

**Results:**

Variants within *APOE *(rs7412, rs429358), *PON1 *(rs854560), *ITGB3 *(rs5918), and *NOS3 *(rs2070744) were found to be associated with one or more blood lipids in at least one race/ethnic group in crude and adjusted analyses. In non-Hispanic whites, no individual polymorphisms were associated with any lipid trait. However, the *PON1 *A-G haplotype was significantly associated with LDL-C and TC. In non-Hispanic blacks, *APOE *variant rs7412 and haplotype T-T were strongly associated with LDL-C and TC; whereas, rs5918 of *ITGB3 *was significantly associated with TG. Several variants and haplotypes of three genes were significantly related to lipids in Mexican Americans: *PON1 *in relation to HDL-C; *APOE *and *NOS3 *in relation to LDL-C; and *APOE *in relation to TC.

**Conclusions:**

We report the significant associations of blood lipids with variants and haplotypes in *APOE*, *ITGB3, NOS3*, and *PON1 *in the three main race/ethnic groups in the U.S. population using a large, nationally representative and population-based sample survey. Results from our study contribute to a growing body of literature identifying key determinants of plasma lipoprotein concentrations and could provide insight into the biological mechanisms underlying serum lipid and cholesterol concentrations.

## Background

Decades of research have demonstrated that serum concentrations of blood lipids are associated with increased risk for cardiovascular disease and mortality [1-4]. Previous reports from the Framingham Heart Study suggested a strong positive relationship between coronary heart disease and elevated levels of total cholesterol (TC) and low-density lipoprotein cholesterol (LDL-C) levels, in addition to an inverse relationship between the disease and high-density lipoprotein cholesterol (HDL-C) levels [5-8]. The genetic basis for elevation in lipid levels is not well understood, but substantial heritability has been demonstrated in twin [9] and family-based [10-12] studies, which have estimated that approximately 43% to 83% of the variance in blood lipid and lipoprotein levels is attributable to genetic factors. Recent candidate gene studies [13-16], as well as genome-wide association studies [17-25], have identified polymorphisms that account for a portion of the variation in blood lipid levels.

Many genes involved in metabolic pathways have been found to contribute to lipid level variability [14,26,27]. However, conflicting findings are common among genetic association studies. Inconsistencies might be caused by differences in study design, study populations (geographic and ethnic background), statistical methods and power, allele frequencies, and gene-environment interactions. It is not clear if such findings are generalizable to the U.S. population. To assess genetic variation among racial and ethnic groups in the U.S. population, we need genetic information from a large, well-designed, and population-based U.S. survey, such as the Third National Health and Nutrition Examination Survey (NHANES III) that includes the three major race/ethnic groups. Therefore, we sought to investigate the associations between 22 polymorphisms in 13 candidate genes and serum lipid concentrations using data from the NHANES III, a nationally-representative survey of the U.S. population.

## Methods

### Study population

NHANES III is a multi-stage complex probability survey conducted from 1988 to 1994 by the National Center for Health Statistics (NCHS) of the Centers for Disease Control and Prevention (CDC) [28,29]. The survey was designed to provide nationally representative statistics on the civilian, non-institutionalized U.S. population aged 2 months or older. A DNA bank was created from blood samples collected during the second phase of NHANES III (1991-1994) from participants aged 12 years or older. This DNA bank provided one of the first opportunities to assess genetic variation among major racial and ethnic groups using a well-designed, population-based, and nationally representative sample of the U.S. population. The bank contains specimens from 7,159 participants, 62% of whom originated from households containing multiple family members (mean: 1.59 participating members per household; range 1-11). More information on the DNA bank is available on the NCHS Web site [30].

We combined genetic data with behavioral, environmental, and clinical information available in NHANES III. We restricted our analyses to participants aged 17 years or older (n = 6,317). Of these, we included only those who self reported as non-Hispanic white, non-Hispanic black, or Mexican American (n = 6,016), who had their blood drawn in the morning (n = 2,712), who fasted at least 9 hours (n = 2,488), and who did not take cholesterol-lowering medications (n = 2,413). This study was approved by the NCHS Ethics Review Board.

### Selection of polymorphisms

We tested 22 polymorphisms in 13 candidate genes that were chosen from a set of variants that we previously genotyped in the NHANES III DNA Bank [31], including polymorphisms in *ABCB1*, *ADH1C*, *ADRB2*, *ADRB3*, *APOE*, *ITGB3*, *MTHFR*, *MTRR*, *NOS3*, *SERPINE1*, *PON1*, *PPARG*, and *TNF *(Additional file 1, Table S1). The candidate genes included in this current study were identified from systematic literature reviews on previously published associations with blood lipid levels, chosen based on the biology of the disease locus in relation to the outcomes, or chosen based on prior linkage studies. Information on the nucleotide or amino acid change for each variant is included in Additional file 1, Table S1.

### Genotyping methods

All genotypes were analyzed using TaqMan (Applied Biosystems, Foster City, California) or MGB Eclipse (Nanogen, Bothell, Washington) assays. Polymorphisms that passed blind-replicate analyses (≥ 98% of genotypes matched) were tested for deviation from Hardy-Weinberg proportions (HWP) using standard chi-square goodness-of-fit tests. Variants that deviated from HWP at *p *< 0.01 for at least two of the three included race/ethnic groups (i.e., non-Hispanic white, non-Hispanic black, and Mexican American) were excluded from further analysis. Detailed genotyping methods and quality control criteria have been previously described [31] or can be obtained from NCHS (in the case of *APOE*).

### Laboratory measures and phenotype definitions

Details of the blood collection procedures and the laboratory evaluation of LDL-C, HDL-C, TC, and TG are available online [32]. Serum LDL-C was calculated using the Friedewald equation [33]. Participants who did not fast, who fasted fewer than 9 hours, or who had TG levels greater than 400 mg/dL were excluded in the analyses.

Phenotypic covariates included in the analyses were previously reported to be associated with blood lipid levels [34]. In non-genetic models, age and body mass index (BMI) were both strongly associated with blood lipid concentrations within each race/ethnic group (Additional file 1, Table S2). The remaining risk factors were significantly associated with at least one lipid measured in at least one race/ethnic group. The covariates included in the final models were: age (17-39 years, 40-59 years, or ≥ 60 years); sex; education completed (less than high school, high school, or college and above); alcohol intake (none, <4 drinks per week, ≥ 4 drinks per week); smoking status (current smoker, former smoker, non-smoker); BMI; physical activity [none, low (active <5 times per week), or high (active ≥ 5 times per week)]; and log of total fat intake (g/day, reported in a dietary recall from the previous 24-hour period).

### Statistical analysis

All analyses were performed using SAS-Callable SUDAAN 9.01 (Research Triangle Institute, Research Triangle Park, North Carolina) and SAS 9.1 (SAS Institute, Cary, North Carolina) to account for the NHANES III complex sampling design. For each genetic variant, univariate and multivariable regression models were used to test for genetic associations with each blood lipid measurement, stratified by self-reported race/ethnicity. Interaction between each variant and race/ethnicity was examined to test racial/ethnic differences in the genetic effects. We assumed an additive model of inheritance and used regression analyses to test the null hypothesis that LDL-C, HDL-C, TC, or TG levels did not differ by an increasing number of minor alleles. Beta-coefficient estimates and 95% confidence intervals for each variant were calculated in regression models using sample weights that were recalculated for the NHANES III DNA bank data. TG levels were log-transformed to approximate a normal distribution.

Haplotype analysis was also performed for each of the seven genes for which at least two polymorphisms were genotyped: *ADH1C, ADRB2, APOE, MTHFR, NOS3, PON1*, and *TNF*. Haplotype frequencies were inferred within each racial/ethnic group using the Expectation-Maximization algorithm [35,36] available in the HAPLOTYPE procedure in SAS/Genetics. The inferred haplotypes with rare frequency (<1%) were combined into one variable ("other"). Haplotype trend regression analyses [37,38] were conducted using crude and multivariable regression models, as described above.

For both the single variant analyses and haplotype analyses, the p-value from Satterthwaite statistics was adjusted to control the false discovery rate (FDR) [39], a method for correcting for multiple testing, in each of the three race/ethnic groups separately. An association was considered significant at an FDR-adjusted p-value of < 0.05.

We used Quanto (University of Southern California, Los Angeles, California; http://hydra.usc.edu/gxe/) to estimate the power of our study. Assuming additive genetic models, we determined the beta-coefficients that correspond to a genetic variant explaining 1% of the variation in the lipid measurements for allele frequencies ranging from 0.01 to 0.5. For these beta-coefficients and allele frequencies, we calculated the lower and upper limits of our power which account for our multiple testing adjustments using the effective sample sizes (sample sizes multiplied by a design effect of 1.2 to account for the complex sampling design of NHANES III) of the three race/ethnicities.

## Results

Characteristics of the participants included in this study are described in Table 1. Non-Hispanic whites (n = 989) were the oldest, had obtained higher levels of education, and were the most physically active compared to non-Hispanic blacks and Mexican Americans. Non-Hispanic blacks (n = 683) were least likely to have consumed any alcoholic drinks in the past week, were most likely to be current smokers, and had the highest mean body mass index (BMI). Mexican Americans (n = 741) were the youngest, had the highest proportion of male participants, and were the least likely to smoke. Blood lipid levels were also significantly different (at *p *< 0.05) across the three main race/ethnic groups. Non-Hispanic blacks tended to have the highest HDL-C levels compared with non-Hispanic whites and Mexican Americans. In contrast, non-Hispanic whites had the highest LDL-C and TC levels; whereas, Mexican Americans had the highest serum triglycerides levels.

**Table 1:** Characteristics of study fasting samples - NHANES III (1991-1994)

Characteristics	Non-Hispanic White (n = 989)		Non-Hispanic Black (n = 683)		Mexican American (n = 741)		P-value**
	N	Weighted*% (SE)	N	Weighted % (SE)	N	Weighted % (SE)	
Age (years)							
17-39	328	45.57 (1.88)	386	55.67 (2.30)	426	65.75 (2.32)	<.0001
40-59	251	31.92 (2.07)	194	29.89 (1.97)	166	25.74 (1.98)	
> = 60	410	22.51 (1.88)	103	14.45 (2.02)	149	8.51 (1.11)	
Sex							
Male	380	47.00 (1.78)	273	43.07 (1.79)	370	51.34 (1.17)	0.0053
Female	609	53.00 (1.78)	410	56.93 (1.79)	371	48.66 (1.17)	
Education							
<High School	246	18.26 (1.60)	225	30.40(2.83)	445	57.56 (2.84)	<.0001
High School	363	35.71 (1.83)	252	37.23 (2.57)	176	25.64 (2.22)	
College and Above	379	46.03 (2.64)	204	32.37 (3.21)	114	16.80 (2.39)	
Physical Activity							
None	195	16.69 (1.47)	199	28.74 (2.60)	256	31.35 (2.11)	<.0001
Low	354	36.29 (1.82)	215	31.07 (1.87)	222	30.30 (1.75)	
High	440	47.02 (2.50)	269	40.19 (2.71)	263	38.35 (2.22)	
Alcohol Intake							
None	536	45.67 (2.63)	375	55.16 (1.75)	376	48.25 (1.72)	0.0268
<4	237	28.82 (1.86)	135	22.00 (1.30)	170	26.66 (2.74)	
> = 4	198	25.51 (1.83)	145	22.84 (1.44)	174	25.09 (1.82)	
Smoking Status							
Current	207	24.40 (2.02)	198	29.87 (1.54)	149	20.39 (1.65)	<.0001
Former	291	28.36 (1.57)	87	13.43 (1.48)	161	19.34 (1.99)	
Non-smoker	491	47.24 (2.36)	398	56.70(1.91)	431	60.26 (1.67)	
	**N**	**Mean (SE)**	**N**	**Mean (SE)**	**N**	**Mean (SE)**	P-value***
	
BMI	989	26.40 (0.20)	683	28.17 (0.28)	739	27.83 (0.25)	<.0001
Total Fat Intake (g/day)****	963	90.21 (2.49)	658	85.63 (2.90)	724	84.35 (2.33)	0.1826
Serum HDL (mg/dL)	983	49.33 (0.78)	675	53.88 (0.97)	739	46.58 (0.67)	0.0001
Serum LDL (mg/dL)	962	126.35 (1.20)	665	122.96 (1.57)	710	118.55 (1.25)	0.007
Total Cholesterol (mg/dL)	985	202.07 (1.46)	677	197.91 (1.39)	739	193.37 (1.49)	0.004
Serum Triglycerides (mg/dL)	984	138.73 (4.82)	677	107.27 (2.36)	738	146.67 (4.29)	0.0001

SE, standard error.

*Frequencies were adjusted using the NHANES III genetic sample weights.

**Chi-square test.

***P-value was calculated using the Satterthwaite-adjusted F test.

****Reported in a dietary recall from the previous 24-hour period.

Allele frequencies for the study variants among the three racial/ethnic groups in the U.S. population are available in Additional file 1, Table S1. Each genetic variant was tested for association with each of the four blood lipid measurements. Table 2 lists the genetic variants with significant associations ((false-discovery rate (FDR)-adjusted p-value < 0.05)) in at least one race/ethnic group for the crude or adjusted regression models. Complete results for all studied variants, with and without FDR adjustment of p-values, are available in Additional file 1, Table S3a-d (crude analyses) and Table S4a-d (covariate-adjusted analyses). The FDR-adjusted and unadjusted p-values for testing racial/ethnic differences in the genetic effects (SNP × race/ethnicity interaction) are also included in these Additional Tables. In fasting samples from non-Hispanic whites, none of the studied variants were found to be significantly associated with any blood lipids after adjustment for multiple testing. For non-Hispanic blacks, *APOE *rs7412 was strongly associated with both LDL-C and TC in crude and adjusted analyses. We observed that several polymorphisms were significantly associated with blood lipids in the Mexican American population, including: *PON1 *rs854560 with HDL-C in both crude and adjusted analyses; *APOE *rs7412 and rs429358 with LDL-C and TC in adjusted analyses; and *NOS3 *rs1799983 with LDL-C in adjusted models only. None of the 22 polymorphisms were found to be associated with triglyceride levels except for *ITGB3 *rs5918 in non-Hispanic blacks.

**Table 2:** Crude and adjusted associations between blood lipids and variants by race/ethnicity - NHANES III (1991-1994)

Genetic Variant	Non-Hispanic White				Non-Hispanic Black				Mexican American				SNP X Race/ethnicity
		
	β (95% CI)			FDR-adjusted P value*	β (95% CI)			FDR-adjusted P value	β (95% CI)			FDR-adjusted P value	FDR-adjusted P value
**Crude Analysis**													
**High-density lipoprotein cholesterol**													
rs854560 (*PON1*)	-2.23 (-5.15,0.69)			0.3589	-0.58 (-2.81,1.65)			0.869	3.59 (1.55,5.64)			0.0308	0.2552
**Low-density lipoprotein cholesterol**													
rs7412 (*APOE*)	-16.68 (-27.85,-5.50)			0.1144	-20.58 (-29.12,-12.04)			<.0001	-19.80 (-33.33,-6.28)			0.066	0.8931
**Total serum cholesterol**													
rs7412 (*APOE*)	-13.90 (-25.39,-2.41)			0.1569	-16.26 (-25.75,-6.77)			0.0374	-10.26 (-19.18,-1.33)			0.2871	0.9227
**Triglycerides**													
rs5918 (*ITGB3*)	0.05 (-0.06,0.17)			0.7294	0.14 (0.07,0.20)			0.0066	-0.05 (-0.17,0.06)			0.613	0.4653
Adjusted Analysis**													
**High-density lipoprotein cholesterol**													
rs854560 (*PON1*)	-1.53 (-4.11,1.06)			0.5062	-0.09 (-2.20,2.02)			0.9733	3.21 (1.45,4.97)			0.022	0.1535
**Low-density lipoprotein cholesterol**													
rs7412 (*APOE*)	-16.87 (-27.94,-5.81)			0.0968	-22.52 (-30.01,-15.04)			<.0001	-21.47 (-31.32,-11.62)			0.0022	0.9012
rs429358 (*APOE*)	8.26 (1.19,15.33)			0.264	3.85 (-2.14,9.84)			0.6169	10.54 (6.41,14.67)			<.0001	0.9012
rs1799983 (*NOS3*)	3.05 (-2.41,8.52)			0.5284	5.28 (-0.42,10.98)			0.4103	-4.56 (-7.63,-1.48)			0.0396	0.9012
**Total serum cholesterol**													
rs7412 (*APOE*)	-15.26 (-26.16,-4.36)			0.0902	-20.68 (-28.90,-12.47)			<.0001	-12.54 (-19.89,-5.20)			0.0198	0.9724
rs429358 (*APOE*)	9.82 (2.96,16.69)			0.0902	4.35 (-2.30,11.01)			0.6839	11.18 (7.11,15.25)			<.0001	0.9724
**Triglycerides**													
rs5918 (*ITGB3*)	0.02 (-0.07,0.11)			0.8985	0.14 (0.09,0.19)			<.0001	-0.01 (-0.14,0.11)			0.8962	0.4419

β, beta coefficient; CI, confidence interval; FDR, false discovery rate

*P-value was based on Satterthwaite-adjusted F test and adjusted for multiple comparisons using false discovery rate within each race/ethnicity group.

**Adjusted for age, sex, education, body mass index, smoking status, alcohol intake, physical activity, dietary fat intake.

Haplotypes with significant associations with blood lipids in at least one race/ethnic group in crude or adjusted models are listed in Table 3. Complete results are included in Additional file 1, Tables S5a-d (crude analyses) and S6a-d (covariate-adjusted analyses). In the three race/ethnic groups, all polymorphisms within a gene were in linkage disequilibrium (*p *< 0.05 from linkage disequilibrium test; data not shown). Consistent with the results of the individual polymorphisms, haplotypes within three genes (*APOE, NOS3*, and *PON1*) were found to be significantly associated (FDR-adjusted p < 0.05) with blood lipid levels. In non-Hispanic whites, the only significant associations found were for the A-G haplotype of *PON1 *in relation to elevated LDL-C in crude and adjusted models. Among non-Hispanic blacks, an inverse association was found in crude and adjusted analyses between the T-T (ε2 isoform) haplotype of *APOE *and LDL-C (*p *≤ 0.0010 for both) and TC (*p *= 0.052 for crude; *p *= 0.0020 for adjusted). In Mexican Americans, haplotypes of *PON1 (*A-A and A-G) were significantly associated with elevated HDL-C; and *NOS3 *haplotype T-T was significantly associated with decreased levels of LDL-C. In addition, carriers of the *APOE *C-C (ε4 isoform) haplotype had significantly increased LDL-C (borderline significant in crude model and strongly significant in adjusted model) and TC (in the adjusted model); whereas, *APOE *T-T (ε2) carriers had significantly decreased levels of LDL-C and TC (in adjusted models only). The A-G haplotype of *ADRB2 *was significantly associated with elevated HDL-C (in crude and adjusted models) among Mexican Americans (see Additional Tables S5a and S6a). However, the confidence intervals were relatively wide. Rare haplotypes (which were combined and coded as "other") of *MTHFR *and *TNF *were associated with blood lipids in at least one race/ethnic group (Additional file 1, Tables S5a-d, S6a-d). There were no common haplotypes significantly associated with TG levels in any race/ethnic group.

**Table 3:** Crude and adjusted haplotype analysis for blood lipid levels by race/ethnicity - NHANES III (1991-1994)

Genetic Variant	Haplotype	Non-Hispanic White				Non-Hispanic Black				Mexican American			
		
		β (95% CI)			FDR-adjusted P value*	β (95% CI)			FDR-adjusted P value	β (95% CI)			FDR-adjusted P value
**Crude Analysis**													
**High-density lipoprotein cholesterol**													
*PON1*	A-A	-6.56 (-13.78,0.66)			0.3814	-0.91 (-8.16,6.35)			0.9641	6.81 (2.89,10.73)			0.0179
	A-G	-7.94 (-25.71,9.83)			0.697	-1.45 (-9.49,6.59)			0.9641	22.04 (4.73,39.34)			0.0777
	T-A	-3.75 (-7.46,-0.05)			0.3814	-0.12 (-5.58,5.34)			0.9641	1.03 (-3.09,5.14)			0.8002
	T-G	REF				REF				REF			
**Low-density lipoprotein cholesterol**													
*APOE*	C-C	12.12 (-4.40,28.64)			0.2499	4.92 (-5.58,15.41)			0.9016	15.43 (5.73,25.14)			0.0494
	T-C	REF				REF				REF			
	T-T	-30.86 (-56.47,-5.25)			0.1066	-40.25 (-57.28,-23.22)			0.001	-38.64 (-67.23,-10.05)			0.0721
*NOS3*	C-G	-6.17 (-19.32,6.98)			0.4971	2.26 (-15.53,20.05)			0.9861	-0.62 (-10.80,9.56)			0.9665
	C-T	3.85 (-11.06,18.76)			0.7161	38.05 (4.28,71.81)			0.1927	0.24 (-11.33,11.80)			0.9665
	T-G	REF				REF				REF			
	T-T	0.82 (-23.44,25.08)			0.9704	2.94 (-11.50,17.37)			0.9861	-20.78 (-34.52,-7.03)			0.0494
*PON1*	A-A	10.91 (-0.74,22.56)			0.1706	4.89 (-12.82,22.60)			0.9861	2.45 (-9.04,13.94)			0.8789
	A-G	46.49 (21.16,71.82)			0.0063	8.91 (-12.85,30.68)			0.9016	6.44 (-25.95,38.84)			0.8789
	T-A	8.98 (-2.58,20.53)			0.247	-1.09 (-11.58,9.40)			0.9861	-2.04 (-8.21,4.13)			0.8789
	T-G	REF				REF				REF			
**Total serum cholesterol**													
*APOE*	C-C	14.28 (-2.27,30.82)			0.2221	6.28 (-4.99,17.54)			0.7536	15.21 (2.29,28.14)			0.3143
	T-C	REF				REF				REF			
	T-T	-24.87 (-51.22,1.48)			0.2172	-31.33 (-50.53,-12.13)			0.052	-19.40 (-38.15,-0.64)			0.3143
Adjusted Analysis**													
**High-density lipoprotein cholesterol**													
*PON1*	A-A	-4.86 (-11.20,1.48)			0.4856	-1.92 (-9.08,5.24)			0.8857	6.14 (3.44,8.84)			0.0021
	A-G	-5.79 (-14.56,2.98)			0.4856	2.17 (-5.89,10.24)			0.8857	19.62 (6.84,32.39)			0.0441
	T-A	-3.22 (-6.60,0.17)			0.4856	1.52 (-4.67,7.71)			0.8857	1.23 (-2.73,5.20)			0.7102
	T-G	REF				REF				REF			
**Low-density lipoprotein cholesterol**													
*APOE*	C-C	12.93 (-3.41,29.26)			0.3459	4.01 (-8.26,16.28)			0.8555	21.28 (13.93,28.64)			<.0001
	T-C	REF				REF				REF			
	T-T	-31.14 (-56.65,-5.64)			0.0992	-44.33 (-59.39,-29.27)			<.0001	-43.23 (-64.13,-22.34)			0.0016
*NOS3*	C-G	-3.42 (-15.49,8.65)			0.676	2.68 (-13.71,19.06)			0.8555	-3.05 (-8.55,2.46)			0.6162
	C-T	6.48 (-10.27,23.22)			0.6032	31.18 (4.64,57.72)			0.233	-0.69 (-10.02,8.64)			0.9344
	T-G	REF				REF				REF			
	T-T	1.64 (-20.75,24.03)			0.8806	1.95 (-10.32,14.21)			0.8555	-32.14 (-47.72,-16.55)			0.0016
*PON1*	A-A	8.77 (-5.24,22.77)			0.4865	8.10 (-9.66,25.85)			0.8555	-2.54 (-15.41,10.32)			0.8479
	A-G	40.03 (19.65,60.41)			0.0053	10.90 (-12.43,34.24)			0.8555	9.62 (-21.64,40.88)			0.7428
	T-A	9.78 (-0.77,20.33)			0.2366	-2.95 (-13.93,8.03)			0.8555	-3.33 (-12.53,5.87)			0.7428
	T-G	REF				REF				REF			
**Total serum cholesterol**													
*APOE*	C-C	16.52 (0.51,32.53)			0.153	5.32 (-8.35,18.99)			0.8062	22.41 (14.73,30.10)			<.0001
	T-C	REF				REF				REF			
	T-T	-27.18 (-52.46,-1.90)			0.153	-40.42 (-57.41,-23.43)			0.002	-25.23 (-39.80,-10.66)			0.0119

β, beta coefficient; CI, confidence interval; REF reference group.

*P-value was based on Satterthwaite-adjusted F test and adjusted for multiple comparisons using false discovery rate within each race/ethnicity group.

**Adjusted for age, sex, education, body mass index, smoking status, alcohol intake, physical activity, dietary fat intake.

## Discussion

In this study, we evaluated statistical associations between blood lipid levels and candidate genes involved in a number of biological pathways, such as nutrient metabolism, immune response and inflammation, oxidative stress, and homeostasis. To our knowledge, there is only one other study (i.e., Keebler et al.) [40] published that describes genetic associations with blood lipid levels using a nationally representative sample of the U.S. population. This study also used data from the NHANES III survey, but associations were examined at 19 genome-wide validated loci on fasting and nonfasting samples. Those data were not available for our use while the present study was being conducted. We examined a different set of polymorphisms which had been identified previously through candidate gene association studies. In our analyses, we used only fasting samples in accordance with guidelines from The National Cholesterol Education Program (NCEP) Expert Panel on Detection, Evaluation, and Treatment of High Blood Cholesterol in Adults (Adult Treatment Panel III, ATPIII) [34].

Our findings suggest, before and after adjustment for numerous demographic and behavioral characteristics in one or more race/ethnic groups, that blood lipid levels differ by an increasing number of minor alleles of polymorphisms in *APOE*, *ITGB3, NOS3*, and *PON1*. Our results also show that the A-G haplotype of *ADRB2 *was associated with elevated HDL-C among Mexican Americans. However, these results from crude and adjusted models are unstable (wide confidence intervals) and would need more data collected to support the association. We found that the group of rare haplotypes (frequency <1%) within *MTHFR *and *TNF *were associated with several blood lipids across race/ethnic groups; but, we are unable to identify which rare haplotype(s) contribute to these findings. Consequently, we cannot interpret these associations.

In analyses of individual variants and of haplotypes, we found strong statistical associations between genetic variation in *APOE *and LDL-C and TC levels in non-Hispanic blacks and Mexican Americans. *APOE*, one of the most studied genes in risk assessment of cardiovascular disease, plays a key role in the metabolism of cholesterol and triglycerides by binding to receptors on the liver and helping to mediate the clearance of chylomicrons and very low-density lipoproteins from the bloodstream [41-46]. Allelic variation in *APOE *has been associated consistently with plasma concentrations of total cholesterol and LDL cholesterol [42,47], and with protein levels of APOB (the major protein of LDL, VLDL, and chylomicrons).

Our findings suggested an association of the *NOS3 *rs1799983 variant and T-T haplotype with LDL-C in Mexican Americans. NOS3 serves as a key enzyme of the endogenous nitrovasodilator system, which is essential for the regulation of vascular function and blood pressure, through the production of nitric oxide. The Glu298Asp variant (rs1799983) has been significantly associated with higher plasma LDL cholesterol, LDL particle size, and lower plasma HDL cholesterol; but no significant associations were found with the T-786C variant [48]. Numerous studies have also reported a positive association with the Glu298Asp variant and haplotypes containing this variant with higher triglycerides and LDL cholesterol in Venezuelans [49] and Greeks [50].

We found higher HDL-C among Mexican American carriers of the PON1 rs854560 (Leu55Met) variant and A-A and A-G haplotypes. Conversely, we found higher LDL-C in non-Hispanic white carriers of the A-G haplotype. *PON1 *is an HDL-associated esterase that hydrolyzes products of lipid peroxidation and prevents the oxidation of HDL and LDL. In fact, the antioxidant activity and anti-atherogenic effect of HDL is thought to be largely because of the paraoxonase located on the HDL particle. Variants in *PON1* previously have been associated with serum HDL and LDL cholesterol levels [51,52], and with increased risk for stroke [53]. There have been multiple studies and meta-analyses evaluating the association of *PON1* variants with blood lipids in several populations or community-based samples, but with inconsistent results [51,54-61].

Our results suggest a strong association of *ITGB3 *with triglycerides in non-Hispanic blacks. ITGB3 is a membrane receptor for fibrinogen and von Willebrand factor that has an important role in platelet aggregation. The Pro33 allele (rs5918) has been associated with coronary thrombosis [62,63] and stroke [64,65]. A previous study examined associations between 15 single nucleotide polymorphisms across *ITGB3 *and cardiovascular disease-related traits in the Hutterites (e.g., plasma levels of HDL and LDL cholesterol and triglycerides) and suggested that *ITGB3 *has sex-specific associations with plasma lipoprotein(a) [66].

Although we did not assess racial/ethnic difference in the genetic effects, we observed that two associations, both involving the rs7412 variant in *APOE*, were significant in two racial/ethnic groups. No variants were significant across all three racial/ethnic groups after the FDR adjustment. Limited power and statistical chance may explain, at least, in part, the lack of consistent findings across the three race/ethnicities. Alternatively, these differences may be caused by varying linkage disequilibrium patterns at causal loci across different race/ethnic populations or by gene-environment interactions that have not been identified or measured. As a result, it might not be unusual to find varying risks for a disease or trait at a given genomic locus across population subgroups. In agreement, a recent study examined 12 newly discovered genetic variants known to predict lipid levels in Europeans and also evaluated local ancestry at validated genes that influence lipid levels [67]. This study found genetic differences between the determinants of lipid phenotypes across different African and European populations. Such findings might suggest that many of the truly causal variants in different race/ethnic groups have yet to be discovered, as most genetic epidemiology studies have been performed in populations of European descent.

Although we identified associations of *APOE*, *ITGB3, NOS3*, and *PON1 *with blood lipid levels by examining polymorphisms individually, our results suggest that assessing genetic variation using haplotype methods might be more comprehensive and more informative. We found that although a single genetic variant might have a small (if any) effect in identifying a susceptibility locus for an outcome, the effect might reach statistical significance when combined with other variants within the gene. For example, after adding a single variant (*APOE *rs7412) to a regression model containing non-genetic risk factors, we were able to explain only slightly more variation in LDL-C (R^2 ^= 0.1448 for non-Hispanic white persons, 0.2065 for non-Hispanic black persons, and 0.1462 for Mexican American persons) compared to the variation explained by non-genetic risk factors alone (R^2 ^= 0.1163, 0.1533, and 0.1230, respectively). However, we observed that a larger proportion of the variation in LDL-C is explained by the model that contains the *APOE *T-T haplotype compared to the model containing the rs7412 variant alone (R^2 ^= 0.1521, 0.2073, and 0.1636, respectively). Overall, the variance in blood lipid levels explained by the contribution of each individual variant or haplotype is considerably small (<5%; data not shown).

The present study has many notable strengths. First, the study was conducted using a large population-based and nationally representative survey of the United States. The wealth of data in NHANES facilitated the examination of genetic, environmental, and clinical data for each of the three major race/ethnicities in the United States. Moreover, whereas many previous reports were limited to a single population or were based on smaller study populations, we were able to conduct the analyses separately in each race/ethnicity, and were therefore able to account for the differences in allele frequencies, disease prevalence, and linkage disequilibrium patterns between these subpopulations. Finally, the control of hypercholesterolemia is an important clinical and public health objective. Awareness of, and screening for, hypercholesterolemia have become more common in recent years. Accordingly, treatment of the condition has increased since the initiation of the National Cholesterol Education Program in 1985. The use of cholesterol-lowering medications has increased steadily in U.S. adults aged ≥ 20 years, from 8.2% in 1999-2000 to 14.0% in 2005-2006, as measured in NHANES [68]. Among those diagnosed with hypercholesterolemia, the proportion on treatment increased from 32.4% to 38.9% in the 8-year period from 1999 to 2006 [69]. Association analyses of genetic variants involved in influencing blood lipid levels may therefore be complicated by a high prevalence of study participants who take lipid-lowering drugs. An advantage of this study in NHANES III is that a small number of participants taking such medication needed to be excluded (n = 75; 3% of fasting samples). Evaluation of such genetic associations in subsequent NHANES surveys will result in a loss of a higher number of participants in the analyses.

In addition to these strengths, we acknowledge several limitations. To help reduce the chance of potential false-positive results from multiple testing, we adjusted p-values to control the false discovery rate [39]. This method assumes that the set of tests are independent. Yet, we know that many of the test statistics might be correlated because of linkage disequilibrium between genetic variants [70]. The FDR adjustment, therefore, might result in overly conservative p-values, thus decreasing our ability to identify true associations.

Although we stratified the analysis by race/ethnicity, we cannot eliminate completely the possibility of confounding of our study results by population stratification. We were not able to assess population structure in our analysis and grouped participants by broad categories on the basis of self-reported race and ethnicity. Substantial admixture in the African American and Hispanic populations has been documented [71-74]. However, previous research conducted on the U.S. population has found little evidence for population substructure in whites [75].

Although the NHANES III data may be more representative of the U.S. population than other non-population-based samples, the statistical power to detect genetic associations was limited in this study. For example, we determined the beta-coefficients that correspond to the genetic variant explaining 1% of the variation in LDL-C. The beta-coefficients ranged from 5.2 to 26.3 depending on the frequency of the minor allele (MAF = 0.01 to 0.5). Using these beta-coefficients and corresponding allele frequencies described above for LDL-C, we found that our power would be 42-82% for non-Hispanic whites, 24-66% for non-Hispanic blacks, and 27-70% for Mexican Americans (Additional file 1, Table S7).

## Conclusions

We report the significant association of blood lipid levels with variants and haplotypes in *APOE*, *ITGB3, NOS3*, and *PON1 *in multiple race/ethnic groups in the United States, using a large, nationally representative and population-based sample survey. Because of strengths of the study design, these findings could be generalized to the U.S. population. Results from our study contribute to a growing body of literature identifying key determinants of plasma lipoprotein concentrations and might provide insight into the biological mechanisms underlying serum lipid and cholesterol concentrations.

## Abbreviations

CDC: Centers for Disease Control and Prevention; CI: confidence interval; FDR: false-discovery rate; HDL-C: high-density lipoprotein cholesterol; LDL-C: low-density lipoprotein cholesterol; MAF: minor allele frequencies; NCHS: National Center for Health Statistics; NHANES III: Third National Health and Nutrition Examination Survey; NOPHG: National Office of Public Health Genomics; SNP: single nucleotide polymorphism; TC: total cholesterol; TG: triglycerides.

## Competing interests

The authors declare that they have no competing interests.

## Authors' contributions

MC led the development of the analytic plan, worked closely with AY in the analyses of the data, interpreted the findings, and drafted the manuscript. AY developed the statistical methods and conducted the data analyses. RMN performed literature reviews, provided genotyping and genetic consultation, and performed editorial review. PWM conceived of the original research question, gave critical input on research methods, and helped to interpret the findings. NFD serves as Team Lead of the Population Health Research Team in the National Office of Public Health Genomics (NOPHG) and oversees the CDC/NCI NHANES III Genomics Working Group. NFD provided scientific leadership for development of the research plan, interpretation of findings, and helped write the manuscript. All authors read and approved the final manuscript.

## Pre-publication history

The pre-publication history for this paper can be accessed here:

http://www.biomedcentral.com/1471-2350/11/62/prepub

## Supplementary Material

Additional file 1 **Table S1.** Allele frequencies by race/ethnicity - NHANES III (1991-1994). The allele frequencies by race/ethnicity are shown. Table S2. P-Values from the Univariate analysis for covariates - NHANES III (1991-1994). The p-values from the univariate analysis of covariates are shown. Table S3a-d. Univariate analysis for HDL-C, LDL-C, TC, and TG, and candidate genetic variants by race/ethnicity - NHANES III (1991-1994). The univariate analysis for lipids and candidate genetic variants by race/ethnicity is shown. Table S4a-d. Association between HDL-C, LDL-C, TC, and TG, and selected genetic variants adjusting for covariates by race/ethnicity - NHANES III (1991-1994). The association, by race/ethnicity, between lipdis and selected genetic variants adjusting for covariates are shown. Table S5a-d. Crude haplotype analysis for HDL-C, LDL-C, TC, and TG, by race/ethnicity - NHANES III (1991-1994). The crude haplotype analysis for lipids by race/ethnicity is shown. Table S6a-d. Adjusted haplotype analysis for HDL-C, LDL-C, TC, and TG, by race/ethnicity - NHANES III (1991-1994). The adjusted haplotype analysis for lipids by race/ethnicity is shown. Table S7. Power calculation by race/ethnicity - NHANES III (1991-1994). Power calculation by race/ethnicity is shown. Click here for file
